# Paired measurement of urinary creatinine in neonates based on a Jaffe and an enzymatic IDMS-traceable assay

**DOI:** 10.1186/1471-2369-15-62

**Published:** 2014-04-15

**Authors:** Karel Allegaert, Pieter Vermeersch, Anne Smits, Djalila Mekahli, Elena Levtchenko, Steven Pauwels

**Affiliations:** 1 Neonatal Intensive Care Unit, University Hospitals Leuven, Herestraat 49, 3000 Leuven, Belgium; 2 Department of Development and Regeneration, KU Leuven, Belgium; 3 Clinical Department of Laboratory Medicine, University Hospitals Leuven, Leuven, Belgium; 4 Department of cardiovascular sciences, KU Leuven, Belgium; 5 Department of Paediatric Nephrology, University Hospitals Leuven, Leuven, Belgium

**Keywords:** Urinary creatinine, Newborn, Jaffe, Enzymatic, IDMS traceable

## Abstract

**Background:**

Urinary creatinine can be quantified by Jaffe or enzymatic assays and is commonly used as denominator of urinary excretion of electrolytes or protein. Paired analysis in pediatric and adult samples documented inter-assay differences (up to 80%). We verified the interchangeability of two IDMS-traceable assays (Jaffe and enzymatic) for neonatal urine and report on neonatal urinary creatinine values using these IDMS-traceable methods.

**Methods:**

Creatinine was measured in 84 neonatal urine samples from 46 neonates by an IDMS traceable Jaffe and enzymatic assay (Roche Diagnostics, Cobas c702 module). Creatinine values, differences in urinary creatinine and clinical characteristics were described and covariates of between assay difference were explored (Wilcoxon, Bland-Altman, correlation, multiple regression).

**Results:**

Median Jaffe and enzymatic urinary creatinine concentrations were 9.25 (range 3.7-42.2) and 9.15 (range 3.8-42.9) mg/dL respectively, resulting in a median difference of 0.08 (SD 0.6, range −2.4 to 0.96) mg/dL. In a multiple regression model, urinary enzymatic creatinine concentration (r = 0.45) and postnatal age (r = −0.59) remained independent variables of the difference between both assays (r^2^ adj = 0.45).

**Conclusions:**

The tested IDMS-traceable assays showed interchangeable in heterogeneous neonatal urine samples. Using these assays, neonatal urinary creatinine showed 5–20 fold lower values than those observed in children or adults with a significant negative correlation with postnatal age.

## Background

Assessment of renal function includes glomerular filtration rate (GFR) and renal tubular transport activities. This necessitates quantification of urinary creatinine, either to calculate creatinine clearance as GFR estimate or as denominator to quantify renal tubular activities (e.g. sodium, potassium, amino acids, proteins, calcium, phosphate, glucose) [1,2]. However, neonates have a lower muscular mass resulting in lower creatinine synthesis. They also display extensive maturational changes in GFR. Both phenomena result in significantly lower median urine creatinine concentrations (9.7 mg/dL) [2] compared to observations in children (50 mg/dL) [3] or adults (154 and 269 mg/dL) [4,5]. In addition, tubular maturation initially results in renal wasting of small solutes and a more limited urinary pH range while diuresis increases in the first days or weeks of life [1,6]. Finally, changes in renal elimination of conjugated bilirubin occur secondary to age-related glucuronidation capacity [6].

For accurate and interchangeable test results adequate standardization is of utmost importance. Historically, creatinine assays showed great variation depending on reaction mechanism (Jaffe or enzymatic reaction) and manufacturer. Consequently, expected creatinine values in neonates depended on the specific method used. IDMS-traceable creatinine assays have been introduced to solve this problem [7]. The IDMS re-calibration is, however, more extensively verified for serum than urine matrix by the manufacturer (internal communication with Roche Diagnostics). This standardization also does not eliminate differences related to the reaction mechanism. Jaffe methods display interference by endogenous (e.g. pseudocreatinines, bilirubin, glucose) and exogenous (e.g. cefalosporins) substances. Although enzymatic methods are less prone, these assays can also be affected by interferences (e.g. bilirubin, dopamine) [8-10]. Due to the above-mentioned maturational changes and frequent medication use, the composition of neonatal urine is variable and different from adults. Given the method specific interferences, urinary creatinine may still vary in an method and assay specific way with between assay differences related to the composition of neonatal urine. Differences up to 80% have been describes in children comparing Jaffe and enzymatic assays, with median urinary concentrations of 69 to 75 mg/dL depending on the method applied [3]. Taking above-mentioned remarks into account, the interchangeability of two IDMS-traceable assays (Jaffe and enzymatic) was verified for neonatal urine. Furthermore, we report on neonatal urinary creatinine values using IDMS-traceable methods.

## Methods

### Clinical procedure and sampling

Urine samples used in this study were initially collected as part of recently reported studies on paracetamol or propylene glycol disposition (propylene glycol containing formulations for intravenous paracetamol, digoxin, phenobarbital, or diphantoine administration) in neonates [11,12]. For both studies, neonates were considered for inclusion after approval of these studies (EUdraCT 2009-011243-39 and B-32220084836 respectively) by the Ethics Board of the University Hospitals Leuven, Belgium and following informed written parental consent. To further reduce the burden, urine collection was only performed in neonates in whom an urinary bladder catheter was already present for clinical indications. After a 6 h time interval, the urine was collected and the volume was recorded. Subsequently, a sample was stored at −20°C until analysis. Clinical characteristics (birth weight, current weight, postnatal age, gestational age) were recorded at the day of sample collection.

### Bio-analysis

Samples were analysed by a Jaffe and an enzymatic method, applying the clinical available analytic methods currently available in the University Hospitals Leuven (Roche Diagnostics Jaffe (urine application without compensation factor and a sample dilution factor of 1:25) and enzymatic method (dilution factor of 1:50), both IDMS traceable, measured on a Cobas c702 module) [13,14]. Total imprecision (expressed as CV) of the Jaffe assay determined according to CLSI EP5 was 2.2% at 61.5 mg/dL and 2.2% at 138.9 mg/dL with a measuring range from 4.2 to 622 mg/dL. For the enzymatic assay, total imprecision was 1.4% at 64.8 mg/dL and 1.2% at 145.8 mg/dL with a measuring range from 1.1 to 610 mg/dL. Internal quality control (iQC) was performed with commercial control material (Biorad unassayed) using simplified Westgard rules for statistical process control and biological critical acceptance limits [15]. As external quality control (eQC) the Biorad Unity urine chemistry report was used. This system allows comparison of our iQC results with values determined in other laboratories using the same method, analyzer and QC lot. We used a difference of more than 2 standard deviations of our mean from the peer group mean as acceptance criterion. For plasma creatinine, we also participated in the mandatory eQC scheme of the Belgian National Scientific Institute of Public Health. At the time of measurement of the study samples, there were no problems with iQC as well as eQC results.

### Statistics

Clinical characteristics and analytical results were described by median and range, mean and standard deviation, or incidence. Passing-Beblok regression and Bland-Altman were performed to explore the difference between both measurements and trends in its variability. Paired analysis (Wilcoxon) was performed to quantify differences between both analyses, and covariates of differences were explored (Rank correlation, multiple linear regression). A p-value < 0.05 was considered statistically significant (MedCalc®, Mariakerke, Belgium). Clinical relevance was evaluated by using desirable biological criteria for creatinine concentration (24 h) for bias (8.6%), imprecision (12.0%) and total error allowable (TEa) (28.4%) [15].

## Results

Eighty-four urine samples were available for analysis. These samples were collected in two cohorts of 23 neonates each who had a median gestational age of 35 (range 24–41) weeks, had a postnatal age of 5 (range 1–26) days, while birth weight was 2 680 (605–4 300) g and weight at inclusion 2 640 (605–4 300) g. The median urine volume (mL/6 h) was 58 (range 5.5-304) mL, equal to 3.7 (range 0.6-11.8) mL/kg/h.

**Figure 1:**
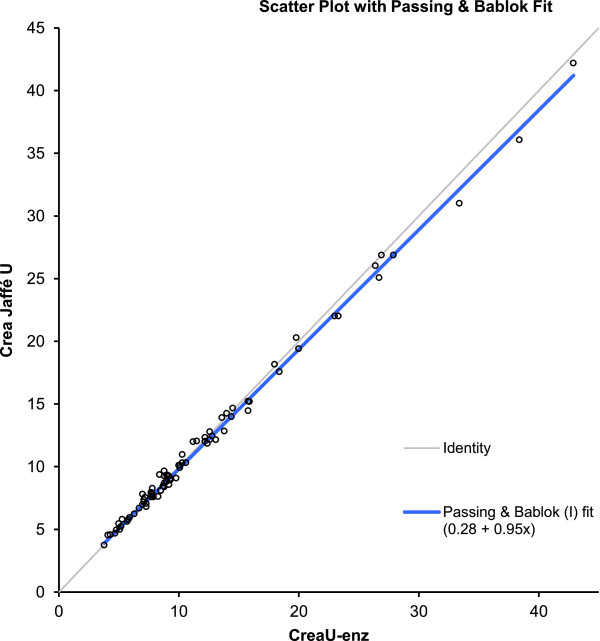
**Passing-Beblok (y = 0.28 + 0.95 ×, 95% CI slope 0.93-0.98, 95% CI intercept 0.12-0.48) between enzymatic and Jaffe creatinine measurements (both mg/dL).** (X-axis = creatinine in urine enzymatic, CreaU-enz, mg/dL; Y-axis = creatinine in urine Jaffe, Crea Jaffe U, mg/dL).

**Figure 2:**
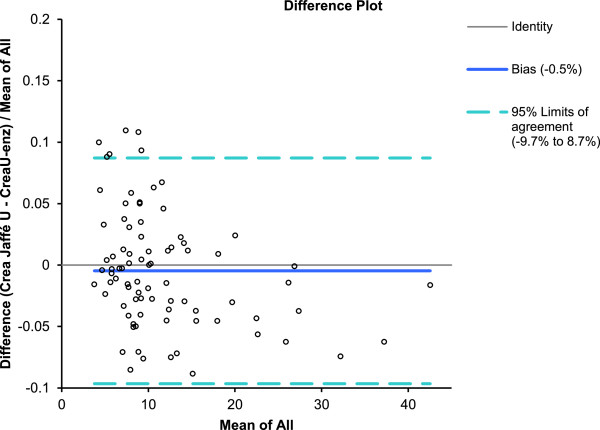
**Bland Altman plot illustrates a mean difference of 0.2 mg/dL, equal to 0.4% in creatinine concentration measured.** There is also an impact of the creatinine concentration on the direction and extent of this difference between both assays. A higher creatinine urine concentration (>25 mg/dL) results in a higher enzymatic compared to a Jaffe assay, while the reverse is true in lower urine concentrations (<10 mg/dL) (X-axis = mean of all, mg/dL; Y-axis = difference [creatine in urine, Jaffe – creatinine in urine, enzymatic/mean of all]).

**Figure 3:**
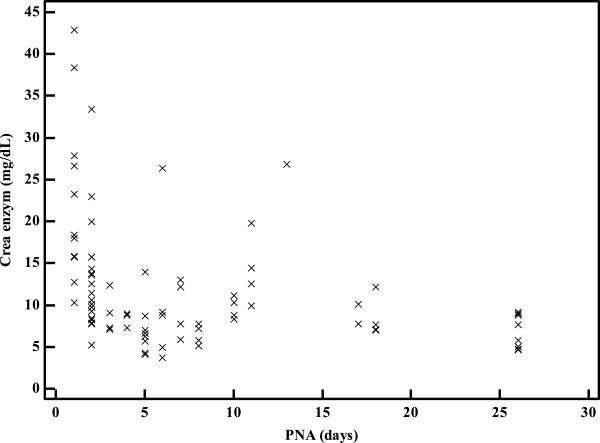
**A significant inverse correlation (r = − 0.59, 95% CI - 0.72 to - 0.43, p < 0.001) between postnatal age (days) and urinary creatinine concentration (enzymatic method, mg/dL) is documented, likely in part reflecting the oliguria and the associated higher urinary creatinine concentrations in early neonatal compared to late neonatal life.** (X-axis = postnatal age (days, PNA); Y-axis = creatinine in urine, enzymatic, mg/dL).

Median IDMS traceable Jaffe urine creatinine was 9.3 (range 3.7 - 42.2) mg/dL, and median IDMS traceable enzymatic urine creatinine was 9.1 (range 3.8 - 42.9) mg/dL, resulting in a median difference (Jaffe – enzymatic) of 0.1 (SD 0.6, range −2.4 to 1.00) mg/dL (p <0.001). A significant Passing-Bablok regression between both creatinine measurements was documented (y = 0.28 + 0.95 ×, 95% CI slope 0.93-0.98, 95% CI intercept 0.12-0.48)) (Figure 1). Bland-Altman further illustrates the mean difference (0.2 mg/dL, equal to 0.4%), but also illustrates that there is a creatinine concentration related impact on both the direction and the magnitude of the difference between both measurements (Figure 2). Finally, there are also significant correlations between the urinary creatinine concentrations and some indicators of maturation (age), most significant for postnatal age (enzymatic method, r = −0.59, 95% CI −0.72 to −0.43, p < 0.001, Figure 3). There were no significant differences between urinary samples from preterm compared to term neonates.

In a multiple regression model, urinary creatinine concentration and postnatal age remained independent variables of the difference between both assays, resulting in a r [2] adjusted value of 0.45 (p < 0.001) (coefficients of the regression equation: constant = −0.2147, creatinine 0.04508 and postnatal age −0.01966 respectively).

## Discussion

Paired measurement of urinary creatinine in neonates based on Jaffe and enzymatic IDMS-traceable assays resulted in a limited mean difference of 0.2 mg/dL. Our regression curve shows that enzymatic measurements are proportionately 4.96% higher than Jaffe measurements. This is well within the predefined biological criteria. All individual paired results were within TEa. As proportionately higher enzymatic values (within biological criteria) were also observed in a method comparison experiment (part of the method validation) using random urine samples from adults, this is probably to a large extent caused by a calibration difference. Our study (and also the method validation), however, gives a snapshot of the difference between methods using specific lots of calibrators and reagents. A new comparison using different lots might yield slightly different results. Based on our results Jaffe values are higher than enzymatic at lower creatinine concentrations. This could in part be explained by a constant positive interference in the Jaffe assay (urinary amino acids, glucose, bilirubin, pseudocreatinines) as no compensation factor is used. In this context, the independent significant effect of PNA on the difference can probably also in part be explained by urinary matrix variation with age and consequential variation in interference with predominantly the Jaffe assay. A limitation of our study is that we did not use direct comparison to a gold standard method to further examine the difference. The manufacturer, however, states traceability to a gold standard method in the insert.

The overall lower creatinine synthesis and clearance capacity in neonates is reflected in a lower urinary creatinine concentration These values are indeed significantly lower (5–20 fold) when compared to values reported in children or adults [3-5,10]. Taking into account these low concentrations and the exponential increase of the CV at these low levels, the determined difference is remarkably low. In a publication of Srivastava *et al.* (comparing a Jaffe with an enzymatic assay, both non-IDMS traceable), differences up to 80% for creatinine urine values in urine samples of 18 children have been reported [3]. For our study, the maximum observed difference was 12%, documented at the lowest creatinine concentrations (Figure 2).

## Conclusions

The tested IDMS-traceable assays showed interchangeable in heterogeneous neonatal urine samples. Using these assays, neonatal urinary creatinine showed 5–20 fold lower values than those observed in children or adults with a significant negative correlation with postnatal age.

## Competing interests

Besides the funding for governmental agencies mentioned below, the authors declare that they have no other competing interests.

## Authors’ contributions

KA conceived of the study, and participated in its design, study registration, sample collection and coordination and drafted the consecutive versions of the paper. PV and SP participated in the design, were responsible for the bio-analysis, and have been involved in drafting the manuscript or revising it critically for important intellectual content. AS, DM and EL have been involved in drafting the manuscript or revising it critically for important intellectual content. All authors read and approved the final manuscript.

## Pre-publication history

The pre-publication history for this paper can be accessed here:

http://www.biomedcentral.com/1471-2369/15/62/prepub
